# Embedded Philanthropic CSR in Digital China: Unified View of Prosocial and Pro-environmental Practices

**DOI:** 10.3389/fpsyg.2021.695468

**Published:** 2021-09-01

**Authors:** Qing Ye, Zain Rafique, Rongting Zhou, Fahad Asmi, Muhammad Azfar Anwar, Ahmad Nabeel Siddiquei

**Affiliations:** ^1^Department of Science and Technology of Communication, University of Science and Technology of China, Hefei, China; ^2^College of Information Engineering, Fuyang Normal University, Fuyang, China; ^3^Key Laboratory of Immersive Media Technology (Anhui Xinhua Media Co., Ltd.), Ministry of Culture and Tourism, Hefei, China; ^4^Department of Governance and Public Policy, National University of Modern Languages, Islamabad, Pakistan; ^5^Research Institute of Business Analytics and Supply Chain Management, College of Management, Shenzhen University, Shenzhen, China; ^6^Department of Management Sciences, COMSATS University Islamabad, Vehari, Pakistan; ^7^Bond Business School, Bond University, Gold Coast, QLD, Australia

**Keywords:** CSR, gamified charity, S-O-R framework, functional transparency, telepresence, warm glow

## Abstract

In recent decades, China has transformed from a conventional society into a digitally competitive nation. From an economic perspective, embedded corporate social responsibility (CSR) is gaining a new height where gamified charity is a trendy approach. By adopting the norm activation model from the point of view of the stimulus–organism–response framework, this research theoretically conceptualized the role of the mobile application environment (including telepresence, functional transparency, and accessibility) to map the cognition and philanthropic behavioral intentions of consumers in the gamified setting. The quantified survey comprised 669 respondents. The findings highlighted the critical role of functional transparency and telepresence of a mobile application in driving consumers’ warm glow and ascribed responsibility. The research underlined the presence of the unique DNA of Internet Plus Charity (Public Benefits) for prosocial and pro-environmental purposes in China under the umbrella of philanthropic CSR.

## Introduction

Corporate social responsibility (CSR) provides a broadly coupled scope of organizations’ responsibilities on the ethical, social, and organizational fronts (Bian et al., 2021). It reflects that shareholder economic benefits must come up in a sustainable manner (Matten and Moon, 2008). Kao et al. (2018) mentioned that CSR practices in mainland China are primarily influenced by present external factors (i.e., being part of the global supply chain) and internal influencers (i.e., institutional code of conduct). Companies in China strive to gain market share while keeping their social and ecological concerns balanced and well addressed. Interestingly, the reputation of non-state-owned enterprises is highly influenced by the degree of social and environmental standards the firms have achieved (Barnea and Rubin, 2010; Zhang et al., 2015). Yu et al. (2017) also indicated that CSR positively promotes a firm’s competitive advantage among competitors. The Chinese government has legalized CSR into a law framework, indicating that CSR is widely accepted in China while in the era of Industry 4.0 (Yu et al., 2017; Kao et al., 2018). In the context of corporations, CSR practices have been recognized as a prominent approach to improve competitiveness; therefore, companies have increasingly chosen to embed social responsibility into their operations and management (Xiao and Yang, 2020) in which the new practice paradigm of corporate philanthropy practice is formed on the basis of the Internet (Xiao et al., 2020).

In mainland China, the concept of philanthropy is evolving while having unique Chinese characteristics, which broadly overlap prosocial and pro-environmental behaviors. Therefore, the combination of charity and public benefits can be interchangeably used for prosocial and pro-environmental issues and concerns. Their core value (service) generally addresses socioecological issues, that is, poverty alleviation, disaster relief, education, ecological and cultural conservation, and science and technology development. Furthermore, information and communications technology (ICT) can be regarded as a catalyst to increase philanthropic practices in society.

The tracking of the fusion of “ICT” and “philanthropy” comprises three stages. First, the Internet cooperates with philanthropy as a communication tool. Second, IT offers a communication function to philanthropy in which IT remains a subsidiary of philanthropy but integrates gradually. Third, the Internet and philanthropy have fully merged as an organic whole called “*Internet Plus Charity (Public Benefits)”* in China. The characteristics of the Internet, such as data, intelligence, cloud, open, interactive, and other IT-driven innovative concepts, have been used to extract the realized potential for philanthropic reforms. As a result, several new charitable participation (public benefit) modes have appeared in the electronic world. Another exciting change is that new models combine virtual and real practices to show the impact of the real world.

In the context of *Internet Plus Charity (Public Benefits)*, the public has various engaging approaches to charity that have features of diversification, contextualization, and gamification, such as donation from reading, walking, a daily low-carbon lifestyle, and even usage or consumption of daily application programs. From the point of view of corporations, it is an emerging paradigm that embeds corporate philanthropy into product and operation; for example, “Ant Forest” by Alipay and “Fulfilling *Dream Elf”* by Toutiao. Such an emerging paradigm is called corporate philanthropic behavior, but also belongs to cause-related marketing. Meanwhile, it offers a convenient way for the public to join charity (public benefits). The current paper coins the term “gamified charity” to highlight the strategic use of games to drive and trigger charitable concerns (specifically, social benefits). That is, the ICT has minimized the effort required by potential initiators/contributors by providing electronic platforms to transform their intentions in a real-world manner. Research examines the potential explanatory power of accessibility (*ACC*), telepresence (*TEL*), and functional transparency (*TRA*).

In particular, the authors take the contextual constructs defined on the basis of application-environment properties to drive the philanthropic behaviors of potential consumers. That is, there is an enormous amount of literature existing on the philanthropic behavioral mapping established by the norm activation model (NAM). However, none of the existing literature highlights the role of virtual application attributes in predicting the philanthropic behaviors of potential consumers. Thus, the present study takes a unique position as the research objective to underline the significance of the attributes of the virtual application environment. *ACC*, *TEL*, and *TRA* while explaining the philanthropic intentions of consumers. Apart from this unique essence of the research, it further expands the mediating role of motivation-driven (warm glow, *WG*) and consequence-driven (ascribed responsibility, *AR*) cognitive aspects as determinants while exploring the relatedness between the attributes of the application environment and consumers’ philanthropic intentions. In the recent literature pool, social and personal norms have been used as exogenous factors to define responsible behavior. To further expand the theoretical implication of the current research, the moderating role of the normative environment (NS) is investigated while determining the endogenous factor (philanthropic behavioral intentions) in mainland China.

## Corporate Philanthropy and Gamified Charity

Corporate social responsibility is a multidimensional construct that includes a range of corporate behaviors aimed at fulfilling the expectations of different stakeholders (Farop et al., 2016; Jia, 2020). In recent times, CSR has been examined in several businesses and economic environments, that is, human resource management (Kao et al., 2018), marketing differentiation (Yu et al., 2017), and portfolio planning and management (Geerts and Dooms, 2020). Furthermore, some studies have emphasized that internal CSR promotes employees’ engagement in voluntary eco-concerned initiatives (Su and Swanson, 2019; Alsuwaidi et al., 2021). Nan and Heo (2013) indicated that cause-related marketing elicits a favorable consumer attitude toward corporate identity. The adoption of ICT is an effective method to effectively communicate CSR to stakeholders/potential participators (Du et al., 2010).

In general, the concept proposed by Carroll (1979) can define CSR, which includes four dimensions: economic, legal, ethical, and philanthropic responsibilities. Philanthropic responsibilities involve actions that connect with society’s expectation (participating in society’s welfare) (Carroll, 1991). As a representative behavior of CSR, corporate philanthropy can transmit the positive social values of an enterprise to internal and external stakeholders, thus improving the corporate image to form a competitive advantage (Zhou et al., 2019). In China, the corporate philanthropy development model has changed from “government-led and enterprise participation” to “Enterprise building platform and multisocial participation” (Xiao et al., 2020). Meanwhile, ICT-driven corporate philanthropy has gained popularity, especially among young people. Therefore, an increasing number of companies have launched gamified charity, which integrates gamified elements with charity to transform its potential value into the realized ones in a convenient manner. Specifically, interactive game design can be a crucial factor in attracting extensive attention and broad participation from the public.

Gamification means implementing game design elements in non-game contexts (Deterding et al., 2011). Gamification can stimulate the willingness of users to participate in any given task or activity (Huotari and Hamari, 2012). That is, gamification aims to enhance assigned charges or actions in an exciting manner (Koivisto and Hamari, 2014). The operating principle of gamification is to employ, engage, and reward users who produce cognitive, persuasive, and psychological effects (Koivisto and Hamari, 2019). Typical gamification forms include points, leaderboards, achievements, feedback, clear goals, and narratives (Hamari and Koivisto, 2015), which can be described as creating social competition and incentivizing behavior through badge and reward systems (Hanus and Fox, 2015).

In recent decades, gamification has been applied in various fields, such as business, marketing, e-Learning, digital healthcare, and individually and socially sustainable behaviors (Koivisto and Hamari, 2014; Robson et al., 2015; Sardi et al., 2017). Meanwhile, CSR can be captioned as a necessity to differentiate in the highly competitive environment and further lead promotional strategies (Chen and Yang, 2017). In the Internet Plus era, CSR is taking on new fronts and challenges. Gamified charity is corporate philanthropy and essentially belongs to cause-related marketing, operated under the “enterprise building platform and multisocial participation” mechanism. It offers a good way for the public to join charity (public benefits), which is taking new heights in terms of quality, interactivity, and excitement. Typical examples of gamified charities are discussed below.

Recently, Alipay’s “Ant Forest” and Toutiao’s “Fulfilling *Dream Elf”* can be captioned as philanthropic gamified versions with Chinese characteristics. In such ICT-based environments, users become involved in charity by engaging in given tasks or activities in a virtual (gamified) setting, allowing users to perform actions and transform them into real-world scenarios. Specifically, in *Ant Forest*, users accumulate green energy from daily pro-environmental activities. When users achieve a certain amount, they can act toward a realized value (users plant virtual trees on the virtual platform, and then real trees will be planted in a specific geographic location by the platform provider) (Yang et al., 2018; Zhang et al., 2020). The game rule is that users are supposed to collect green energy manually by touching their mobile phone screens before it expires. Attributes are also offered to share the obtained green energy with friends or take it from others while having certain game rules and allowing for the planting of trees with other friends’ help (Wang and Yao, 2020). In addition, the leaderboards show the achievements of users with other competitors in a gamified environment. Moreover, users can receive electronic certificates when they grow trees successfully. Users can also see real trees that are user-grown by using the map function at any time.

*Fulfilling Dream Elf* has a similar game design element. If users have completed specific tasks or activities relevant to the usage of Toutiao, such as daily logins, reading papers, watching videos, searching, or using other functions, the interface of *Fulfilling Dream Elf* generates a certain number of energy points. Users must collect green energy manually before it expires. When users accumulate a certain number of energy points, they can donate books in a virtual setting and transform them into real ones for underprivileged children with the help of the application provider (Toutiao).

## Theoretical Framework and Hypothesis Development

Gamified charity is an emerging corporate philanthropy approach, which is based on digital space and presented in the form of gamification. The operation mechanism is “Enterprise building platform and multisocial participation.” To some extent, gamified charities inherit the fundamental characteristics of social network games (SNGs). Various studies on SNGs across differential theoretical frameworks exist. For instance, Hsu and Lu (2004) extended the Technology Acceptance Model to predict the acceptance of online games by users. Shin and Shin (2011) integrated cognitive and affective attitudes as the acceptance model of SNGs. Wei and Lu (2014) examined the intention of mobile social games on the basis of network externalities and theory of uses and gratifications (U&G). Huang et al. (2016) also integrated U&G and customer engagement as a theoretical stance. Baabdullah (2018, 2020) revised the modified Unified Theory of Acceptance and Use of Technology as a conceptual model to explain the intention to use mobile SNGs. Moreover, Liu et al. (2016) adopted cognitive dissonance theory to explain how the dynamics of abundance of choices alter consumers’ perceptions of their current e-service choices. However, all of the studies discussed above have interpreted the intention of using SNGs from different perspectives. According to Bagozzi (2007), the above-discussed theoretical stance still restricts the intervention of external variables and computation complexities. The challenging view of Bagozzi (2007) escalates its complexity, especially in the case of Information System (IS) research where virtual environment-based constructs are considered for SNGs.

In terms of human–computer interaction, interactivity (as an attribute) can be captioned as the dominating factor to define the attractiveness, involvement, and human cognition of users (Liu, 2017). Therefore, interactivity can also be counted as a construct to map the well-being of users during the experience of human–machine interaction (de Bellis and Johar, 2020). In the gamified environment, increased enjoyment (as a core of the flow experience) defines the cognitive performance of users and improves their prosocial trait and behavior. Apart from the role of immersive experience in terms of value and experience, its influence on human cognition as a research area is still evolving in the literature (i.e., virtuous cycle) (Shin, 2018), thereby heightening users’ experience, related consumption, and related decision processes (Hibbeln et al., 2017). Shin (2017) concluded that the usability and learnability through immersion are directly influenced by their affordance. Similarly, Zhu et al. (2015) and Shin et al. (2016) argued that the perceived value of users and the interactive features of immersive media define their acceptance and usability. D’Errico et al. (2019) emphasized that the virtual world and its high pace of evolution have transformed the lives and perspectives of humans in recent years. For example, the affective relevance of the digital revolution has drastically revolutionized human-computer interaction as it offers affective and empathic interfaces and digital solutions for humans. Within the pool of literature on immersive media interactivity, D’Errico et al. (2020) stated that a high degree of interactivity minimizes the psychological distance between the user and machine. Such a degree also helps to further amplify emotional arousal during the interactive experience. In addition, cognition is often referred to as perspective taking, which allows an observer to extend the affective state, emotional regulation, and prosocial behavior in the virtual space (Papapicco, 2020).

To achieve the primary objectives of this study, the persuasive behavioral and psychological view is adopted. Specifically, the Stimulus Organism Response (SOR) framework by Mehrabian and Russell (1974) is employed, which has been intensively used to explain and investigate the interaction between the environment (external factors), cognition (internal characteristics), and behavioral responses. Jacoby (2002) introduced S–O–R in the field of consumer behavior. The S–O–R framework consists of three basic components, namely, stimulus, organism, and response. Specifically, *stimulus* refers to environmental cues or external elements that influence an individual whose emotional, cognitive, or mental state of *organism* is aroused; it further takes special responsibility as a part of an internal cognitive or a psychological activity (Lin and Lo, 2016; Kamboj et al., 2018). The S–O–R framework has been widely used to research and predict behaviors while mapping entrepreneurial behavior (Liu, 2018), virtual reality tourism behavior (Kim et al., 2018), branding co-creation behavior (Kamboj et al., 2018), and knowledge-sharing behavior (Jia and Xiong, 2020). Moreover, Tang et al. (2019) explored green behavior from the SOR perspective. Participating charity behavior can also be explained by the framework (Qian et al., 2019).

### Stimulus

In the context of the S-O-R framework, exogenous factors stimulate internal cognitive or psychological activities (organism), leading to a certain response. This research proposes three prominent gamified charity characteristics (*ACC*, *TEL*, and *TRA*) as stimuli. These features offer constructs driven from the application environment to predict gamified charity participatory intention (*GCPI*).

Accessibility refers to the degree of ease or convenience with which individuals can access information (Park et al., 2009; Al-debei, 2014). According to existing literature, *ACC* can be captioned as a dominant determinant that impacts the adoption of information systems or application programs, such as the satisfaction of e-banking (Liébana-Cabanillas et al., 2013), the acceptance of the digital library system (Park et al., 2009), and behavioral intention to use e-learning (Revythi and Tselios, 2019). On the contrary, the lack of *ACC* significantly influences the adoption of massive open online courses (Ma and Lee, 2018). Furthermore, Qian et al. (2019) indicated that *ACC* is a critical factor for participating in charitable activities (microcharity). Hence, the study proposes the following hypotheses:

Hypothesis 1 (H1) (a and b): *ACC* influences user *WG* and *AR* in a gamified environment.

Telepresence can be defined as “the *experience of presence in an environment using a communication medium*,*”* which was coined by Steuer (1992). Presence means the natural perception of an environment, while *TEL* emphasizes the sense of “being there” in the phenomenal environment created by a medium (Kim and Biocca, 1997). Fiore et al. (2005) indicated that *TEL* can be understood as an immersive response in which users experience the artificial environment provided with the necessary cognitive or sensory input. Mollen and Wilson (2010) redefined *TEL* as “*a psychological state of being there in a computer-mediated environment, enhanced by focused attention.”* Liu et al. (2020) mentioned that individuals perceive authentic experience from the mediated environment.

Although not a kind of physical or face-to-face presence, *TEL* has been accepted as equivalent to that of direct, embodied experience (Hutchins, 2011). Éric Pelet et al. (2017) also presented that *TEL* is a subjective feeling of immersion in a mediated environment, as a specific immersion case. *TEL* can essentially eliminate temporal or spatial restrictions in a large way. Thus, it is adopted in various business scenarios or product strategies, such as in an online retailer setting (Fiore et al., 2005; Suh and Chang, 2006), e-commerce setting (Lim and Ayyagari, 2018), in the hospitality industry (Ongsakul et al., 2019), social media use (Éric Pelet et al., 2017), and video games (Liu et al., 2020). Moreover, Algharabat et al. (2018) discovered that *TEL* impacts the brands of non-profit organizations, which promotes electronic word of mouth and willingness to donate. Thus, the study hypothesizes that *TEL* in gamified charities affects *WG* and *AR of the participants.*

Hypothesis 2 (H2) (a and b): *TEL* influences user *WG* and *AR* in a gamified environment.

Meanwhile, *TRA* addresses the degree of clear view about processes and system flow. From the public administration and business management perspective, *TRA* means the degree of convenience with which external stakeholders can obtain corporate information (Bushman et al., 2004; Holland et al., 2018). That is, *TRA* refers to the openness flow of information (Bernstein, 2012). Schnackenberg and Tomlinson (2016) proposed that *TRA* entails disclosure, clarity, and accuracy. It can also be maximized by improving visibility and access to open information, great truthfulness, and information accuracy, and reducing information concealment (Yang, 2018).

The literature argues that *TRA* can be persuasive in nature to readers, especially in a philanthropic context where the general evaluation criterion is the degree of transparency. The credibility of a non-profit organization can be promoted by information transparency (Lovejoy and Saxton, 2012). Qian et al. (2019) proved that information transparency is a strategic tool for non-profit organizations to develop public relations. In addition, Cabedo et al. (2018) expressed that great transparency can help the rest of the stakeholders (not only donors) to appreciate the extent to which corporate initiatives contribute to achieving the mission of an organization. Therefore, the study proposes the following hypotheses:

Hypothesis 3 (H3) (a and b): *TRA* influences user *WG* and *AR* in a gamified environment.

### Organism and Response

According to Russell (2009), the psychological state can be described as an effect, an emotion, a mood, and a feeling. He also coined the core effect as a subjective feeling and comprises two underlying dimensions of pleasure–displeasure and activation– deactivation, which can be fused in an integral whole. The structural description of the core effect explicitly matches two dichotomies of internal states in the S–O–R framework: pleasure and arousal–non-arousal (Song et al., 2021). *GCPI* can be divided into motivation-driven (*WG*) and consequence-driven (*AR*) factors as cognitive aspects.

Motivation for prosocial and pro-environmental behaviors can be aroused by the internal affections of individuals, such as seeking pleasure and pursuing happiness (Hartmann et al., 2017). Moral satisfaction can kindly drive motivation, namely, *WG* (Isen, 1970). *WG*, which originated in economics, has been adopted in various fields (e.g., green consumption) (Giebelhausen et al., 2016). It means that subjects can obtain emotional rewards by prosocial and pro-environmental behaviors (Andreoni, 1990, 1995; Taufik et al., 2015; Giebelhausen et al., 2016; Welsch et al., 2021). Jia and van Der Linden (2020) indicated that *WG* in society can drive green behavior. Moreover, Hartmann et al. (2017) found that *WG* significantly affects prosocial behavior more than altruistic value because *WG* is a psychological reward and is readily accepted by adults.

Hypothesis 4 (H4): *WG* influences user *GCPI*.

The degree of prosocial behavior of an individual is usually affected by the degree of their morality. *AR* can be interpreted as individuals who spontaneously lean toward taking responsibility for the consequences of their behaviors (Schwartz, 1977). *AR* is an integral part of NAM proposed by Schwartz (1977), which underlines the feeling of responsibility for adverse consequences caused by following non-prosocial behavior (Bamberg and Schmidt, 2003; De Groot and Steg, 2009; Klöckner, 2013; Han et al., 2020).

Many researchers have confirmed the significant relationship between *AR* and prosocial or pro-environmental behavior; for example, automobile usage behavior (Bamberg and Schmidt, 2003), transport mode change (Nordfjærn et al., 2019), energy consumption practice (Xu et al., 2020), and related pro-environmental behavior (He and Zhan, 2018; Verma et al., 2019; Han et al., 2020).

Hypothesis 5 (H5): *AR* influences the user’s *GCPI*.

### Mediating Role of the Organism

From the perspective of S-O-R, the organism provides a justification for bringing together stimulus and response. In this study, the affective, emotional, cognitive, or mental state of an organism is considered. Mainly, *WG* refers to emotional rewards by prosocial and pro-environmental behaviors (Andreoni, 1990), whereas *AR* reflects the feeling of responsibility for adverse consequences, potentially the outcome of non-prosocial behavior (Schwartz, 1977). The literature references *WG* as endogenous in the cases of *ACC* (Abbott et al., 2013), *TEL* (Hartmann and Apaolaza-Ibáñez, 2012), and *TRA* (Lin et al., 2017). Moreover, *WG* has been investigated while underlining its connection with the pro-environmental intention (Giebelhausen et al., 2016). Similarly, *AR* has also been observed, while emphasizing its connection with the proposed *stimulus* (Nordfjærn et al., 2019). As the mediating effect of attitude, *AR*, and personal moral norm is uncovered (Han et al., 2020), the study proposes that *WG* and *AR* have a mediating role in examining the relationship between the presented stimulus and *GCPI*.

Hypothesis 6 (H6): *WG* mediates the relationship between the proposed set of stimuli (*ACC*, *TEL*, and *TRA*) and the GCPI of users.
Hypothesis 7 (H7): *AR* mediates the relationship between the proposed set of stimuli (*ACC*, *TEL*, and *TRA*) and the GCPI of the users.

### Moderating Role of NS

From the perspective of institutional theory, the behaviors of individuals and organizations can be influenced by a surrounded environment (Sambharya and Musteen, 2014). The normative pillar is a crucial attribute of institutional theory, apart from regulatory and cognitive aspects (Scott, 1995). *NS* can be labeled as “organizational and individual behavior guiding” (Bruton et al., 2010). That is, normative systems are constituted by societal values (what is admired or attractive) and societal norms (what behavior is socially acceptable or how things are done), which generate fundamental rules that individuals and organizations should conform and recognize (Valdez and Richardson, 2013). *NS* reflects specific social values in a given society. In the context of collectivist cultures, individuals tend to respond positively to their community and be easily affected by others, especially in the case of trendsetters, opinion leaders, and prominent members of society (Furnham et al., 2012; Shi et al., 2017).

The literature argues that *NS* has been adopted in several research fields, such as technology adoption (Teo et al., 2003; Liang et al., 2007), green innovation (Aguilera-Caracuel and Ortiz-de-Mandojana, 2013; Berrone et al., 2013), and entrepreneurial activity (Valdez and Richardson, 2013). Moreover, Urban and Kujinga (2017) suggested that *NS* significantly influences social entrepreneurship intentions, which are further examined in green initiatives and pro-environmental concerns by Sreen and Gleim (2020).

Hypothesis 8 (H8) (a and b): *NS* moderates the relationship between the proposed organism (*WG* and *AR*) and the GCPI of the users.

## Methodology

To achieve the goal of the proposed model, we collected data with the help of an online data collection portal. We approached potential respondents through social networking sites (WeChat). The research analyzed the intention toward public willingness to participate in philanthropy (public benefit) electronically. The study proposed the role of attributes of mobile applications (*ACC*, *TEL*, and *TRA*), which can be classified as attributes of human–machine interaction in the gamified environment to define its impact on human cognition (*AR* and *WG*) (H1–3) and further its effect on the intention to use such a digital environment for philanthropic charity (H4 and H5). In addition, the research hypothesized the role of *NS* while mapping the intention of users to use such a virtual environment. This section discusses the instruments adopted, data collection, and analyses.

### Instrument

To address the instruments and the internal and external validity of the study, the instruments for each proposed construct were adopted from the existing literature. Specifically, *ACC*, as a construct, was adopted from the study of Park et al. (2011). Three item constructs for *TEL* were taken from Zhao et al. (2020). Moreover, the three-item constructs for *TRA* were adopted from the research work of Asmi et al. (2019). To examine the organism as a part of S–O–R, *WG* and *AR* were examined on the basis of the three element constructs adopted from Hartmann et al. (2017) and Zhang et al. (2019), respectively. Moreover, normative support as moderator and intention as an endogenous construct were examined by adopting the three item constructs proposed by Venkatesh and Davis (2000), Wei and Lu (2014), and Urban and Kujinga (2017). Each of the construct-related items was measured using the five-point Likert scale and listed in the table below. The Chinese version of the instruments is provided in Appendix A and is backtranslated, following the suggestion of Ye et al. (2020).

### Sampling and Data Collecting

After finalizing the first version of the data collection instruments, the pilot study was conducted involving 20 potential respondents recruited from a university. Members of the pilot study provided valuable suggestions. The revised instrument (questionnaire) included other control variables (e.g., age, gender, and education). The final version of the questionnaire was distributed among potential respondents through social networking sites in the third and fourth quarters of 2020. Because the research involved human participants, the instrument was reviewed and approved by the Ethical Committee of the Department of Science and Technology of Communication - University of Science and Technology of China. Each of the potential respondents communicated about the confidentiality of the information and privacy. Specifically, potential respondents approached and inquired to participate in the survey (as briefly provided in the cover letter). To increase the response rate, follow-up messages were sent to potential respondents, resulting in a total of 669 complete responses, which accounted for further analysis. The descriptive findings are listed in Table 1.

**TABLE 1:** Descriptive profile of the data collected.

**Descriptive**	**Detail**	**Frequency**	**Percentage**
Age	Under 20 20 to 30 30 to 40 Above 40	235 241 104 89	35.13 36.02 15.55 13.30
Gender	Male Female	418 251	62.48 37.52
Intentions to use gamified environment	Entertainment Socialization Others	362 177 130	54.11 26.46 19.43
Average time spent in a gamified environment	Less than 30 min 30–45 min More than 45 min	583 86 –	87.14 12.86 –
Which charitable APP you frequently use (choose only one)	AntForest Fulfilling Dream Elf Others	485 197 14	68.46 29.45 02.09

## Analysis

To examine the proposed model based on the collected data, the authors employed SPSS statistics and ADANCO 2.1 to calculate the confirmatory factor analysis (CFA) and regression to calculate the impact of moderation of the proposed setting. Specifically, the partial least squares (PLS) method was adopted to test the model and hypotheses. PLS enables structural equation modeling (SEM) to test model fitness, composite model, and factor constructs in a unified manner (Henseler et al., 2016). ADANCO, as a statistical tool, provides advanced variance-based SEM support to compute loadings, significance weights, and path coefficients.

### Measurement Model

The authors used ADANCO 2.1 to compute the CFA and the loadings of the item scores to determine the internal and external validity and reliability of the model and the collected data. Favorable scores of factor loadings can be seen in the current data, as loadings are recorded over the continuum of 0.906 and 0.987. To determine the fitness indices of the proposed and saturated model, the SRMR, d_*ULS*_, and dG scores were calculated with the help of ADANCO. All fitness scores were recorded within the acceptable range of values, as suggested by Henseler (2017). The tabular result of the fitness indices is provided in Appendix B.

Convergence reliability was examined by analyzing the loadings of each item, the Cronbach alpha scores and the average extracted variances (AVE); its composite reliability was also considered. The results concluded that the loadings of each item were above 0.01, which is acceptable according to Hair et al. (2014). Furthermore, the lower cut in Cronbach alpha and composite reliability was examined, following the suggestion of a previous pool of literature [see, e.g., Hair et al. (2014)]. The least scores of AVEs were also calculated, which were all observed above 0.50, as recommended by Fornell and Larcker (1981). Thus, the study can be captioned as free of the risks related to the internal reliability of the constructs. The confirmatory factor analysis scores in tabular format are listed in Appendix A.

To analyze external validity, the authors adopted two approaches, as suggested by Hair et al. (2014) and Henseler et al. (2014). Specifically, heterotrait and monotrait (HTMT) were analyzed in which scores lower than 0.850 in the case of each cross-construct relation were taken as upper cut-off points of the acceptable HTMT scores, as recommended by Henseler et al. (2016). Moreover, Fornell and Larcker’s (1981) approach was considered where the correlation scores of each construct were compared with the square roots of the AVEs of the constructs. Acceptable internal reliability was observed in the case of the current investigation. Tabular results are provided in Appendix D.

Furthermore, the extracted variance was examined to avoid the multicollinearity issue, as it can challenge the external reliability of the constructs; the acceptable range of VIF scores was calculated and is shown in Appendix A. Thus, in this study there is no risk of multicollinearity. To extend the scope of instrument and construct validity, the common method biases were calculated following Harman’s single-factor analysis, as advised by Podsakoff et al. (2003) who suggested examining the maximum variance extracted by a single construct. In the present study, the maximum variance observed by a single factor was not more than 23.52%, which can help exclude the risk of common method biases.

### Proposed Model

**FIGURE 1:**
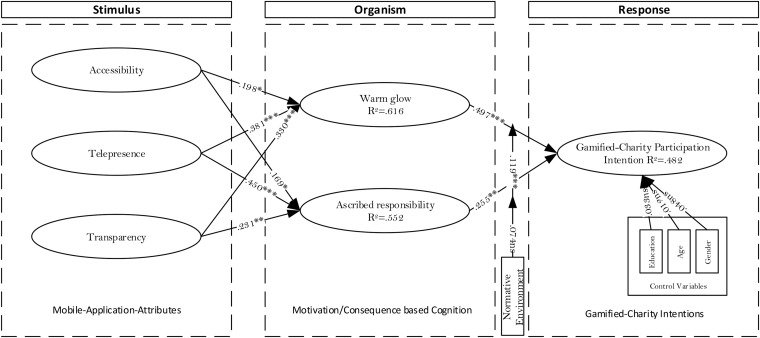
Path analysis of the proposed model.

The results based on our proposed model are indicated in Figure 1. The variance extracted in the cases of *WG*, *AR*, and *GCPI* was observed as 61.6, 55.2, and 48.2%, respectively. The findings revealed that *ACC* has a significant positive impact on *WG* [H1(a): β = 0.198; ρ ≤ 0.05] and *AR* [H1(b): β = 0.169; ρ ≤ 0.05]. A similar trend can be observed in the studies by Qian et al. (2019) and Trond et al. (2019). While examining the role of *TEL*, the findings suggest that it can be captioned as the strongest stimulus among the proposed constructs. In particular, the explanatory power, while defining *WG* and *AR*, was observed as [H2(a): β = 0.381; ρ ≤ 0.001] and [H2(b): β = 0.450; ρ ≤ 0.001], respectively. This result supports the findings recorded by Lewis et al. (2016) and Holdack et al. (2020).

In contrast to other proposed stimuli, *TRA* was observed to be a moderate influencer while highlighting its impact on *WG* [H3(a): β = 0.331; ρ ≤ 0.001] and *AR* [H3(b): β = 0.231; ρ ≤ 0.01], as illustrated in Figure 1. The findings are in line with the results obtained by Cabedo et al. (2018). The impacts of *WG* and *AR* as exogenous factors to underline *GCPI* were recorded as significant. Specifically, the impact of *WG* (H4: β = 0.497; ρ ≤ 0.001) was observed to be more prominent than that of *AR* (H5: β = 0.255; ρ ≤ 0.01). However, none of the control variables (age, gender, and education) were recorded as significant. The results are listed in Table 2.

**TABLE 2:** Path analysis.

	**Effect**	**Original coefficient**	**Standard bootstrap results**				**Cohen’s f^2^**	**Effect size**
			**Mean value**	**Standard error**	***t*-value**	***p*-value (2-sided)**		
H1(a)	ACC − > WG	0.198	0.196	0.107	1.847	0.044	0.065	Medium
H1(b)	ACC − > AR	0.169	0.170	0.082	2.045	0.041	0.041	Weak-medium
H2(a)	TEP – > WG	0.381	0.378	0.104	3.634	0.000	0.180	Medium
H2(b)	TEP – > AR	0.450	0.446	0.099	4.535	0.000	0.215	Medium-Strong
H3(a)	TRA – > WG	0.330	0.332	0.115	2.853	0.004	0.147	Medium
H3(b)	TRA – > AR	0.231	0.229	0.083	2.754	0.006	0.062	Medium
H4	WG – > GCPI	0.497	0.499	0.103	4.824	0.000	0.265	Medium-strong
H5	AR – > GCPI	0.255	0.252	0.092	2.750	0.006	0.069	Medium

To examine the mediation impact of *WG* and *AR* while underlining the relationship between stimuli (*ACC*, *TEL*, and *TRA*) and their relationship to *GCPI*, the method suggested by Preacher and Hayes (2008) was adopted. Specifically, the PROCESS macro was used to perform the mediation analysis. Bootstrapping was also performed with a sample size of 10,000 to compute the asymmetric confidence intervals. The tabular result of the bootstrapping can be seen in Table 3. Specifically, complete mediation was observed in the case of *TRA*. Furthermore, partial mediation was recorded in the cases of *ACC* and *TEL*.

**TABLE 3:** Mediation analysis (bootstrapping results).

**Hypo**	**IV**	**M**	**DV**	**Effect of IV on M**	**Effect of M on DV**	**Direct (c’)**	Indirect (a*b)	**Total effect (c)**	**95% (Cl)**	**Mediation**
H6(a)	ACC	WG	GCPI	0.715***	0.376***	0.411***	0.269***	0.789***	(0.062, 0.466)	Partial
H6(b)	TEL	WG	GCPI	0.696***	0.358***	0.332***	0.249***	0.663***	(0.083, 0.438)	Partial
H6(c)	TRA	WG	GCPI	0.660***	0.491***	0.038ns	0.324***	0.509***	(0.158, 0.512)	Full
H7(a)	ACC	AR	GCPI	0.642***	0.172***	0.411***	0.110***	0.789***	(0.039, 0.274)	Partial
H7(b)	TEL	AR	GCPI	0.658***	0.125***	0.332***	0.083***	0.663***	(0.036, 0.201)	Partial
H7(c)	TRA	AR	GCPI	0.574***	0.257***	0.038ns	0.147***	0.509***	(0.035, 0.304)	Full

*** = Significance level of 0.001.

**FIGURE 2:**
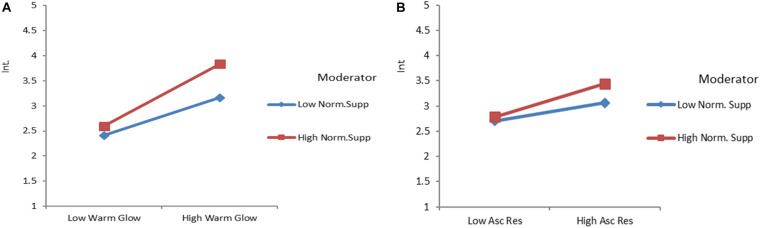
**(A)** Interaction plot of Warm glow and normative support interaction plot **(B)** responsibility assignment and normative support while looking at gamified charity participation intentions.

To analyze the moderation effect of *NS* in the cases of *WG* and *AR* while defining *GCPI*, hierarchal regression analysis was performed. The findings concluded that *NS* strengthens the relationship between *WG* and *GCPI* [H8(a): β = 0.119; ρ ≤ 0.001]. However, a non-significant impact was recorded while underlining the relationship between *AR* and *GCPI* [H8(b): β = 0.074; ρ ≥ 0.05]. The graphical interaction of the moderating effect is shown in Figure 2. In addition, the tabulation details are listed in Appendix C.

## Discussion and Implication

Our descriptive findings conclude that the gamified setting is attractive among young people and is mostly perceived by men as trendy. Most gamified charity participants use such an application environment for entertainment and excitement seeking. The research comprises several theoretical and practical implications, which are important to discuss in detail. The empirical results highlight the critical role of immersive presence and transparency of gamified charity and execution of philanthropic claims offered by the application provider while examining user acceptance and adoption, as suggested by previous literature (Davis et al., 1992; Viswanath, 2000). The literature argues that it has significant potential to predict the willingness of users to transform efforts from the potential value to the realized value (Lewis et al., 2016). However, none of the previous literature underlined *TEL* as an explanatory power simultaneously on motivation-driven (*WG*) and consequence-driven (*AR*) factors. Therefore, the study incorporated the NAM-based S-O-R framework, where stimuli based on attributes of the mobile application were considered. This aspect can be claimed as a unique contribution to IS research.

Furthermore, *TRA* has been considered an exogenous factor to map synchronization / harmonization between critical stakeholders in corporate settings (Holland et al., 2018). *TRA* also affects users in technology adoption and rejection (Mensah, 2018). However, it is never used as an exogenous factor to underline the intention of individuals toward gamified charity. Therefore, our research determined whether *TRA* would influence public intention in gamified charity, which potentially maximizes the effectiveness of corporate philanthropy in the Internet Plus era. In addition, *TRA* is noted as a fully mediated construct by *WG* and *AR*, which enriches the role of *TRA* and highlights the need to examine it further. That is, the proposed model emphasizes the strategic role of *TRA* in the case of a philanthropic application environment as it has dynamic behavior in the motivation-driven (*WG*) and consequence-driven (*AR*) factors.

In addition, *NS* strengthens the relationship between *WG* and *GCPI*. The result confirms that normative pressure can be classified as a strong determinant for restricting or guiding individuals’ behaviors in the context of gamified charity, which can be claimed following previous research (Urban and Kujinga, 2017; Sreen and Gleim, 2020). By contrast, a non-significant relationship is observed between *AR* and *GCPI*, which can further lead to the gap in public understanding and framing issues. Therefore, smart, holistic, and realistic communication strategies are required. In a quantified manner, communication strategies can adopt immersive media (media-rich environment) to communicate and use real-life scenarios, which can help form effective framing strategies where society can quantify their carbon footprints more realistically than before.

Furthermore, gamified charity is corporate philanthropy and essentially belongs to cause-related marketing, operated under the “enterprise building platform and multi-social participation” mechanism, which is trendy, especially among the young generation in China. From a public perspective, gamified charity is a modern way to join philanthropy. It can also be regarded as the adoption of a new Internet application program and the participation of a recent style-based prosocial activity. Our research constructed an integrated model on the basis of the SOR framework to map public intention in gamified charities, which can be considered as a novel contribution in terms of the application of the theoretical framework.

Environment communication research argues that several initiatives struggle to communicate due to the deficiency in the message (Bertolotti and Catellani, 2014), medium (Ettinger et al., 2021; White, 2021) or communication style (Villar, 2021). In the context of risk communication, for the urge to have input from citizens, an effective framing strategy is core to its success (Qiu et al., 2020). The current research initiative highlights the need to use a gamified environment for effective crisis communication, awareness of ecological concerns, and improvement of public ecological footprint.

Several studies have highlighted the importance of information transparency, or *TRA*. However, in our research, *TRA* was examined as an external simulative factor that led to a significant positive change in society and public behavior. Following this view, the degree of transparency of gamified charity decides public perception; thus, greater transparency can maximize public participation chances. Gamified charity has also faced severe ethical challenges, namely prosocial washing and skewed information sharing with stakeholders. Redefining the marketing communication mix is a new need for time, as a hyperactive competitive digital medium demands smart communication tools and tactics.

In the interactive / interactivity grid of users and the nature of the environment, the gamified charity can be captioned as a playful interactive setting to increase public understanding and participation and redefine perception. Our research underlines the unique participatory intention of gamified charities. It emphasizes philanthropic behavior by maximizing the sense of purpose and its relationship to potential participants. The study is individual, as it further expands the view of Marczewski’s Hexad model (Tondello et al., 2016). Participants in gamified charity seem to have motivations to be philanthropists, socializers, free-spirited, achievers, and/or players. However, *ACC*, *TEL*, and *TRA* can be an integrated mobilizer to drive the change in the behavior of participants by provoking prosocial and pro-environmental values. Furthermore, as a description of *Ant Forest* and *Fulfilling Dream Elf* above, the design elements of gamified charity were observed on the basis of points/rewards, leader boards, achievements, feedback, clear goals, and narratives (Hamari and Koivisto, 2015), which can be described as the creation of social competition and the incentivizing of behavior through badge and reward systems (Hanus and Fox, 2015). Therefore, our study signifies that social attributes and reward systems can enhance the core competitiveness of *GCPI*.

Meanwhile, corporate philanthropy should adopt different geographically variated ecological concerns, such as the water crisis in a specific geographic region (water crisis can be considered), to drive philanthropic concerns and awareness. However, in another region, philanthropic issues must be addressed (air pollution or poverty problem) to drive public philanthropic intentions.

The current research environment emphasizes prosocial and pro-environmental activities operated by the “enterprise building platform and multisocial participation” mechanism, which needs legal, social and political support and attention to avoid unpleasant circumstances, such as public distrust in these initiatives. To strengthen the value impact of such initiatives, acknowledgment from macrolevel institutions is highly required.

## Conclusion and Future Research

Gamified charity is the new paradigm of corporate philanthropy practice based on the Internet, which offers a convenient way for the public to join charity (public benefits). That is, gamified charity is formed on the basis of Chinese characteristics embedded into the DNA of Internet Plus Charity (Public Benefits) with prosocial and pro-environmental purposes. The study observes that the gamified environment plays a strategic role in the new paradigm of practice. It shows that the attributes of the philanthropic game-based application environment (including *TRA*, interactive graphical user interfaces, and *ACC*) lead to the philanthropic intentions of individuals. In addition, NAM-based factors, as part of the organism (SOR), mediate the relationship between gamified attributes and charitable intentions. However, further research can be conducted to compare the prosocial and pro-environmental attributes of the citizens in a distinctive way. The belief in efficacy and public understanding of philanthropic issues must also be addressed. Our study is unique as it underlines the significance of Chinese characteristics in participatory philanthropic intentions. These intentions in conventional (traditional) settings can be compared in future research. Corporate philanthropy, as one dimension of the CSR pyramid (Carroll, 1991), has occupied a priority position because of its high rate of visibility and return. CSR in China is still evolving as it is mainly influenced by external actors in the supply chain network. That is, other components of the CSR pyramid should integrate with the world. Therefore, Chinese CSR practices need more attention from researchers and policymakers, especially in the case of Industry 4.0, where personal norms and CSR have a significant relationship.

## Data Availability Statement

The raw data supporting the conclusions of this article will be made available by the authors, without undue reservation.

## Ethics Statement

The study was reviewed and approved by the Ethical Committee of the Department of Science and Technology of Communication – University of Science and Technology of China (STC-USTC).

## Author Contributions

RZ and ZR: concept, initialization, and funding. AS: data collection. FA and MA: data analysis. QY: write-up.

## Conflict of Interest

QY and FA were affiliated to company Anhui Xinhua Media Co., Ltd. as part-time researchers. RZ was a consultant for the company Anhui Xinhua Media Co., Ltd. The remaining authors declare that the research was conducted in the absence of any commercial or financial relationships that could be construed as a potential conflict of interest.

## Publisher’s Note

All claims expressed in this article are solely those of the authors and do not necessarily represent those of their affiliated organizations, or those of the publisher, the editors and the reviewers. Any product that may be evaluated in this article, or claim that may be made by its manufacturer, is not guaranteed or endorsed by the publisher.
