# Supplementary material for: Embedded Philanthropic CSR in Digital China: Unified View of Prosocial and Pro-environmental Practices
Source: Front Psychol. 2021 Sep 1;12:695468. doi: 10.3389/fpsyg.2021.695468 (PMC8440801; doi:10.3389/fpsyg.2021.695468)
Supplement: Supplementary file 1 [file Data_Sheet_1.docx]

Appendix A

Table 1: Confirmatory factor analysis and convergent reliability testing.

| **Construct** | **Item** | **Loadings** | **P_A_** | **P_C_** | **Alpha** | **CReliability** | **Avg.Variance.Ext** |
| --- | --- | --- | --- | --- | --- | --- | --- |
| Accessibility | ACC1  ACC2  ACC3 | .977  .963  .984 | .976 | .982 | .973 | .983 | .950 |
| Telepresence | TEL1  TEL2  TEL3 | .986  .988  .983 | .986 | .990 | .985 | .990 | .972 |
| Functionality | TRA1  TRA2  TRA3 | .969  .984  .980 | .977 | .984 | .976 | .985 | .956 |
| Worm Glow | WG1  WG2  WG3 | .972  .987  .979 | .979 | .986 | .978 | .986 | .959 |
| Ascription of Responsibility | AR1  AR2  AR3 | .965  .960  .939 | .955 | .968 | .973 | .969 | .911 |
| Normative Support | NS1  NS2  NS3  NS4 | .949  .913  .945  .906 | .962 | .961 | .961 | .962 | .862 |
| Gamified Charity Participation Intentions | GCPI1  GCPI2  GCPI3 | .970  .980  .952 | .966 | .977 | .965 | .978 | .936 |

Table 2: Adapted Instruments.

| Constructs | Items | Sources |
| --- | --- | --- |
| 可及性  （Accessibility） | 游戏化慈善平台提供了多种参与公益慈善的方式。  通过游戏化慈善平台，很容易参与公益慈善。  通过游戏化慈善平台参与公益慈善非常方便。 | (Park et al., 2011) |
| 临场感  （Telepresence） | 游戏化公益慈善平台使我觉得自己像是置身于“虚拟世界”，而不是的“现实世界”。  当我使用游戏化公益慈善平台时，我体验到了临场感。  当我使用游戏化公益慈善平台时，我感觉自己处于“虚拟现实”中。 | (Zhao et al., 2020) |
| 功能透明感  (Functional transparency) | 我很容易理解游戏化公益慈善平台的功能。  在访问了游戏化公益慈善平台后，我对它很熟悉。  在访问了游戏化公益慈善平台后，我对它提供的服务非常清楚。 | (Asmi et al., 2019) |
| 暖光效应  (Worm Glow) | 通过参与游戏化公益慈善，我可以体验到愉快的个人满足感。  参与游戏化公益慈善，为社会和自然环境做出贡献，我感到很开心。  参与游戏化公益慈善，让我感到满足，回馈社会和环境。 | (Hartmann et al., 2017) |
| 责任归属  (Ascribed Responsibility) | 我觉得我对人类造成的社会问题和环境恶化负有共同责任  我觉得我对人类造成的社会问题和环境恶化负有部分责任  我相信每个人都对人类造成的社会问题和环境恶化负有部分责任。 | (Zhang et al., 2019) |
| 规范环境  (Normative Support) | 参与游戏化公益慈善在中国非常的流行。  在中国，承担慈善责任被视为成功企业的标志。  这个国家的公众非常认同那些履行慈善责任的企业。 | (Urban & Kujinga, 2017) |
| 游戏化公益慈善参与意图  (Intentions） | 我将继续参与游戏化公益慈善。  我有强烈的意愿在未来参与游戏化公益慈善。  我将尽自己最大努力为社会和自然环境做出贡献。 | (Venkatesh & Davis, 2000) |
| Note:a cover letter about the brief scope, purpose and consent shared with each potential respondent. | | |

Appendix B

Table 3: Fitness indices.

| Estimated Model |  | Score | HI95 | Conclusion |
| --- | --- | --- | --- | --- |
|  | SRMR | 0.0831 | 0.2216 | Supported |
|  | d_ULS_ | 2.2439 | 39.1511 | Supported |
|  | d_G_ | 1.7655 | 21.5280 | Supported |

Appendix C

Table 4: Moderating roles.

| **Hypotheses** | **Hypotheses** | **Significance** | **Beta (Coefficient)** | **Result** |
| --- | --- | --- | --- | --- |
| **H9(a)** | **NS*WG** | p≤.001 | .119 | Supported |
| **H9(b)** | **NS*AR** | p≥.05 | .074 | Not supported |

Appendix D

Table 5(a): Hetro-trait Mono-trait discriminant reliability testing.

| Construct | NS | TEL | TRA | WG | ACC | AR | GCPI |
| --- | --- | --- | --- | --- | --- | --- | --- |
| Normative Support |  |  |  |  |  |  |  |
| Telepresence | .495 |  |  |  |  |  |  |
| Functionality | .392 | .687 |  |  |  |  |  |
| Warm Glow | .461 | .729 | .704 |  |  |  |  |
| Accessibility | .369 | .584 | .529 | .599 |  |  |  |
| Ascription of Responsibility | .437 | .724 | .643 | .689 | .565 |  |  |
| Gam.Char.Part.Int | .487 | .683 | .534 | .686 | .650 | .610 |  |

Table 5(b): Fornell and Larcker's method for discriminant reliability testing

| Construct | NS | TEL | TRA | WG | ACC | AR | GCPI |
| --- | --- | --- | --- | --- | --- | --- | --- |
| Normative Support | **.928** |  |  |  |  |  |  |
| Telepresence | .241 | **.985** |  |  |  |  |  |
| Functionality | .150 | .455 | **.977** |  |  |  |  |
| Warm Glow | .208 | .513 | .474 | **.979** |  |  |  |
| Accessibility | .133 | .328 | .267 | .343 | **.975** |  |  |
| Ascription of Responsibility | .182 | .493 | .386 | .444 | .298 | **.954** |  |
| Gam.Char.Part.Int | .229 | .445 | .269 | .445 | .398 | .344 | **.967** |
| Squared correlations; AVE in the diagonal. | | | | | | | |
